# Thyroid Hormone T3 Counteracts STZ Induced Diabetes in Mouse

**DOI:** 10.1371/journal.pone.0019839

**Published:** 2011-05-27

**Authors:** Cecilia Verga Falzacappa, Claudia Mangialardo, Luca Madaro, Danilo Ranieri, Lorenzo Lupoi, Antonio Stigliano, Maria Rosaria Torrisi, Marina Bouchè, Vincenzo Toscano, Silvia Misiti

**Affiliations:** 1 Dipartimento di Medicina Sperimentale, Sapienza Università di Roma, Roma, Italy; 2 DEM, Fondazione per lo studio del Diabete, l'Endocrinologia ed il Metabolismo, Roma, Italy; 3 Dipartimento di Istologia ed Embriologia Medica, Sapienza Università di Roma, Roma, Italy; 4 Dipartimento di Medicina Molecolare, Sapienza Università di Roma, Roma, Italy; 5 Stabilimento di Utilizzazione di animali da laboratorio, Università Cattolica del Sacro Cuore, Roma, Italy; Florida International University, United States of America

## Abstract

This study intended to demonstrate that the thyroid hormone T3 counteracts the onset of a Streptozotocin (STZ) induced diabetes in wild type mice. To test our hypothesis diabetes has been induced in Balb/c male mice by multiple low dose Streptozotocin injection; and a group of mice was contemporaneously injected with T3. After 48 h mice were tested for glucose tolerance test, insulin serum levels and then sacrified. Whole pancreata were utilized for morphological and biochemical analyses, while protein extracts and RNA were utilized for expression analyses of specific molecules. The results showed that islets from T3 treated mice were comparable to age- and sex-matched control, untreated mice in number, shape, dimension, consistency, ultrastructure, insulin and glucagon levels, Tunel positivity and caspases activation, while all the cited parameters and molecules were altered by STZ alone. The T3-induced pro survival effect was associated with a strong increase in phosphorylated Akt. Moreover, T3 administration prevented the STZ-dependent alterations in glucose blood level, both during fasting and after glucose challenge, as well as in insulin serum level. In conclusion we demonstrated that T3 could act as a protective factor against STZ induced diabetes.

## Introduction

Pancreatic β cell loss is a key factor in the pathogenesis of both type 1 and type 2 diabetes. Whereas in type 1 diabetes β cell destruction is caused by an autoimmune process, type 2 diabetes results from a combination of insulin resistance and impaired β cell function and survival [1], [2]. One of the main processes involved in the insulin-producing β-cell death is apoptosis, which leads to insulin deficiency. Therefore, it is conceivable that a valuable approach to treat or even to prevent the onset of type 1 diabetes, may imply an anti-apoptotic pro-survival therapy of β cells.

Despite major efforts to halt this epidemic and find a cure for diabetes, and even though the critical role of β cell apoptosis in the development and progression of diabetes has been recognized, current treatment strategies do not include the preservation of endogenous β cell mass [3]. Novel approaches able to promote pancreatic β cell reserve and to thwart apoptotic β cell loss are therefore urgently needed.

The entire β cell mass is dictated by a dynamic balance of neogenesis, proliferation, cell size and apoptosis [4]. The IRS/PI3K pathway plays a critical role in the regulation of β cell mass and the Akt kinase is one of the most promising downstream molecules of this pathway, which could be targeted to induce proliferation and survival of β cells [5]. Akt kinase is the principal mediator of the insulin effects on glucose metabolism. It is stimulated by a variety of growth factors, including insulin itself. Studies on mice lacking all the Akt isoforms have highlighted the importance of the Akt1 isoform on the others in the β cell mass [6]. Moreover, the specific overexpression of a constitutively active form of Akt 1 in β cells in mice induces increment in both the size and the number of islet cells [7], [8]; in addition, glucose tolerance is improved and the animals are protected against Streptozotocin-induced diabetes.

*In vitro* experiments in insulinoma cell lines and in isolated islets demonstrated that Akt activation by glucose, insulin, insulin growth factor (IGF-1) and GLP1 is a key event in the anti-apoptotic effects of these molecules [9], [10], [11]. Over the past decade most components in the insulin signalling pathway have been identified in murine and human pancreatic β cells. Insulin signalling has been reported to positively regulate many effects in β cells such as insulin gene expression, insulin secretion, proinsulin byosinthesis and cell cycle progression [12]; the same effects are regulated by glucose. Even the modulation of tribble 3 (TRB3), a cytoplasmic inhibitor of Akt kinase, altered susceptibility to high glucose and ER stress induced apoptosis in INS-1 cells, streghtening the relevance of Akt regulation in β cell mass and function response [13], [14].

Thyroid hormones are widely known for their ability to influence various cellular processes such as mitogenesis and differentiation, which are both considered good candidate targets for counteracting the insorgence of diabetes [15]. We have previously demonstrated [16] that the thyroid hormone T3 stimulates pancreatic ductal cells, considered as β cells precursor, towards a β cell-like phenotype. In addition, we showed [17], [18], [19] that T3 acts as a mitogenic, pro-survival factor in pancreatic β cells, and that it can directly activate Akt; taken together, these results demonstrated that T3 can activate cellular processes strictly related to β cell function such as cell proliferation and survival, cell size regulation, protein synthesis and insulin production. Moreover, our recent study demonstrated that T3 can be a survival factor even for cultured rat islets, counteracting both physiological and pharmacological β cell death. Even in this case T3 can also act as a mitogenic factor [20]. These data strongly sustain our hypothesis that the thyroid hormone T3 can be considered a promoting factor for β cell function, and outline its possible role in contrasting the onset of diabetes.

Based on these data, in this study we intended to verify whether T3 is able to preserve and protect functional β cell mass in STZ diabetic animals.

## Materials and Methods

### Animal studies

Six-weeks-old male Balb/c mice were maintained in a pathogen-free environment in isolator caging system in air conditioned room at 23±1°C in the Cattolica University's animal facilities (Rome, Italy) in accordance to the institutional guidelines.

### Induction of diabetes

Animals were divided into three experimental groups: CTR, STZ, STZ+T3. Diabetes was induced by multiple (for 2 consecutive days) low dose (150 mg/kg of body weight) intraperitoneal injection of Streptozotocin (STZ) (Sigma-Aldrich, Saint Louis, Missouri, USA), freshly dissolved in 10 mM Na-citrate buffer (pH 4,5). STZ-T3 mice were divided into 3 groups and co-treated with 3 different doses of 3,5,3′-Triiodothyronine (T3) (75–100–150 µg of kg body weight)(Sigma-Aldrich) every 24 hours for 2 consecutive days. The T3 treatment began contemporary to Streptozotocin.

### Evaluation of tissue and immunostaining

Mice were anesthetized with ketamine (70 mg/kg) and domitor (1 mg/kg) injected intra peritoneally and then sacrified by cervical dislocation. Pancreata were removed and embedded in killik cryostat embedding medium (Bio-optica, Milan, Italy). Five sections (7 µm thick) per pancreata were examined. Cross sectional islet area was determined on a total of five slides *per* pancreas stained with hematoxilin/eosin. Images were analysed using the Image J software.

For immunohistochemical analysis cryosections were fixed in cold acetone for 1 minute, air-dried and fixed in 4% paraformaldehyde for 10 minutes. Endogenus peroxidase activity was blocked by incubating slides in a solution of 3% hydrogen peroxide for 10 minutes. Unspecific binding was blocked incubating the slides in 5% goat serum in PBS Ca-Mg Free for 45 minutes and then for 15 minutes in 1% BSA. Sections were incubated in humidified chambers overnight at 4°C with the appropriate primary antibody (insulin 1∶100, pAkt 1∶50, Akt 1∶50, Cell Signalling) followed by 1 h incubation with secondary biotinylated anti-mouse or anti-rabbit (Vector Laboratories, Inc., 30 Ingold Road, Burlingame, CA) then the sections were incubated for an additional hour with Horseadish Peroxidase Avidin D (Vector Lab.). Immunoreactivity was revealed using 3,3′-diamonobenzidine (DAB, DAKO, north America, Carpinteria, CA) as the chromogen. Sections were counterstained with hematoxylin.

Terminal deoxynucleotidyl transferase-mediated dUTP nick end-labeling TUNEL assay for evaluation of apoptosis was performed by using *In situ cell death detection kit POD* (Roche, Basel, CH) following manufacturer's instructions. Sections were fixed and incubated for endogenous peroxidase as described previously and then permeabilizated in Triton 0,1% in sodium citrate 0,1% for 2 minutes on ice, washed and air-dried. Sections were incubated 1 hour at 37°C in dark with Tunel reaction mixture, then washed and air-dried. Slides were then incubated with Converted-POD in a humidified chamber for 30 min at 37°C and for 5 min with DAB substrate. Sections were counterstained with hematoxylin.

For immunofluorescence analysis, slides were fixed and blocked as described previously. Primary antibodies (insulin and glucagon, Cell Signalling, 1∶100 and Glut-2, Santa Cruz, 1∶50) were incubated for 1 h at room temperature in humidified chambers. After 3 washes in BSA 1% in CMF slides were incubated with secondary antibodies (Polyclonal swine anti-rabbit Immunoglobulin FITC coniugated, DAKO, Denmark, 1∶200) for 1 h at room temperature in dark. Nuclei were counter-stained with 1 µg/ml Hoechst dye diluited in CMF.

### Measurement of insulin mRna by Real Time PCR

Pancreata RNA was extracted by SV Total RNA isolation System (Promega, 2800 Woods Hollow Road Madison, WI) under manufacture's instruction and 1 µg used to synthetize cDNA using Omniscript RT Kit (Qiagen, Chatsworth, CA). cDNA corresponding to 20 ng of total RNA was used to perform fluorescent-based real-time PCR quantification using the LightCycler Realtime PCR apparatus (Roche Inc., Nutley, NJ). Quantitative PCR was performed using SYBR Premix Ex TAq II (perfect Real Time)(TAKARA BIO INC. 2 Avenue du President Kennedy, 78100 St Germain en Laye, France) as described by the manufacturer. The reactions started with a denaturation step at 95°C for 10 seconds, followed by annealing at 56° to 66°C for 5 seconds and elongation at 72°C for 7 to 13 seconds. The reaction was then heated for 3 seconds at 2°C lower than the melting temperature of the DNA fragment. Oligoprimer pairs that allow the amplification of ∼200 bp were designed and their specificity was verified by blasting in the GenBank database. Reading of the fluorescence signal was taken at the end of the heating to avoid non-specific signal. A melting curve was performed to assess non-specific signal. mRNA expression levels are expressed as number of copies/µg total RNA using a standard curve of the crossing point vs. logarithm of the quantity. The standard curve was established using known cDNA amounts of 0, 10^2^, 10^3^, 10^4^, 10^5^, and 10^6^ copies of 18s and a LightCycler 3.5 program provided by the manufacturer (Roche Inc.). PCR products were analyzed on a 1,5% agarose gel. The expression levels of target genes were quantified and normalized by 18S level. Primers used for PCR amplification were for INS for: 5′-TGGCTTCTTCTACACACCCA-3′ and rev:5′- TGCAGTAGTTCTCCAGCTGG-3′ and for 18S for: 5′-GGAGAGGGAGCCTGAGAAA-3′ and 18S rev: 5′-CGAAAGAGTCCTGTATTGTTATTTT-3′.

### Caspase activity assay

The caspGLOW™ red active caspase staining kit (Biovision Middlefield Way, CA) was used to quantify caspase activity. Freshly-prepared seven-micrometer cryosections were incubated with a Red-VAD-FMK at 1∶300 diluition in PBS Ca-Mg free at 37°C for 45 min and washed twice with the provided washing buffer for 10 min. Nuclei were counter-stained with 1 µg/ml Hoechst dye diluted in PBS.

### Western blot analysis

For total protein extraction, pancreata were removed and immediately freezed in liquid nitrogen. Once freezed tissues were mechanically homogenized and then lysed for 10 min in ice-cold lysis buffer containing 1% Tween 20, 10% glycerol, 150 mmol/L NaCl, 50 mmol/L HEPES pH 7, 1 mmol/L MgCl_2_, 1 mmol/L CaCl_2_, 1 mmol/L NaF, 10 mmol/L Na_4_P_2_O_7_, 2 mmol/L NaVO_3_, 1 mmol/L phenylmethylsulfonylfluoride, protease inhibitors. The lysates were sonicated and centrifuged at 12.000 rpm for 30 min. and the total cellular protein content was measured using Bradford method (Bio-Rad, Richmond, CA, USA). 100 µg of total extract per sample were loaded onto a 10% SDS-polyacrilamide gel, electrophoresed, and then blotted onto nitrocellulose membranes (Bio-Rad). Filters were blocked for non specific reactivity by incubation for 1 h at RT in 5% non–fat dry milk dissolved in PBS 1×, 0,1% Tween 20 and incubated for 16 hours at 4°C with the appropriate primary antibody. Antibodies: anti-Akt and anti-pSer473Akt (Cell Signalling, Danvers, MA, USA, 1∶1000), anti-Glut-2 (Santa Cruz 1∶500), anti Caspase 3 (Millipore,290 Concord Road, Billerica,MA, USA, 1∶200), anti Bax (Santa Cruz 1∶250), anti Tra/b (Santa Cruz 1∶200).

At the end the membranes were washed and incubated for 45 min with the appropriate HRP-conjugated secondary antibody (anti-mouse, anti-rabbit; Sigma-Aldrich 1∶4000) in milk 5%, PBS 1×, Tween 20 0,1% for 45 min at RT.

Immunoreactivity was visualized by the ECL immune-detection system (Amersham Corp, Arlington Heights, IL) in according to manufacturer's instructions. The relative band intensity was evaluated by densitometric analysis (Image J, Wayne Rusband, National Institute of Health, USA) and normalized to total B-actin.

### Trasmission electron microscopy

Tissue fragments were fixed with 2% glutaraldehyde in PBS for 2 h at 4°C. Samples were postfixed in 1% osmium tetroxide in veronal acetate buffer (pH 7.4) for 1 h at 25°C and stained with uranyl acetate (5 mg/ml) for 1 h at 25°C, dehydrated in acetone, and embedded in Epon 812. Ultra-thin sections were examined unstained or poststained with uranyl acetate and lead hydroxide under a Morgagni 268D electron microscope (Fei, Hillsboro, OR, USA).

### Measurements of biochemical parametres

All measurements were performed after 8 h fast.

IPGTT. Glucose tolerance test was carried out 48 hours after STZ and T3 treatment by an intraperitoneal injection of glucose (3 g/kg of body weight) to overnight fasted mice. Glucose and insulin concentrations were determinated in tail vein blood at 0, 30 and 120 min after glucose injection by using Ascenzia Breeze (Bayer AG,51368 Leverkusen, Germany) and Mercodia ultrasensitive mouse insulin ELISA kit (Mercodia, Uppsala, Sweden).

ITT. Mice received an intraperitoneal insulin injection (0,75 U/Kg of bodyweight) under fast condition. Blood glucose concentration was determinated at 0, 30 and 120 min after injection by using Ascenzia Breeze (Bayer AG,51368 Leverkusen, Germany).

### RNA isolation and RT-PCR analysis

Total cellular RNA was isolated from livers by using SV Total RNA Isolation kit (Promega, Madison, WI), according to manufacturer's instructions. RNA (1 µg) was subjected to reverse transcription (RT) by using a cDNA synthesis kit OmniScript (QIAGEN, Chatsworth, CA). cDNA was amplified to determine desiodase I expression using the following primer pairs : (mDioI) 5′-AAGAGGCTCTGGGTGCTCTTGG-3′ and 5′-GGTTCTGGTGATTTCTGATGTC-3′. Amplification was performed for 30 cycles, after a first denaturing step at 94°C for 5 min, at a denaturing temperature of 94°C for 30 sec, at annealing temperature of 55°C for 30 sec and at an extension temperature of 72°C for 30 sec. PCR products were electrophoresed onto a 1.5% agarose gel containing ethidium bromide (0.5 mg/ml) and visualized under UV light. 18s expression levels were analyzed as a control for RNA quality using the following primers for the amplification 5′-GGAGAGGGAGCCTGAGAAA-3′ and: 5′-CGAAAGAGTCCTGTATTGTTATTTT-3′.The relative intensity of the bands was quantitated by densitometric analysis (ImageJ, Wayne Rusband, National Instutite of health, USA) and normalized to the co-amplified 18s cDNA fragments.

All primers were synthesized by MWG Oligo Synthesis Report (Eurofins MWG Operon, Edersberg, Germany)

### Statistical analysis

Different statistical analyses were performed depending on the experimental type and are indicated in the relative Figure legends.

## Results

### T3 treatment preserves islets morphology and dimension

**Figure 1:**
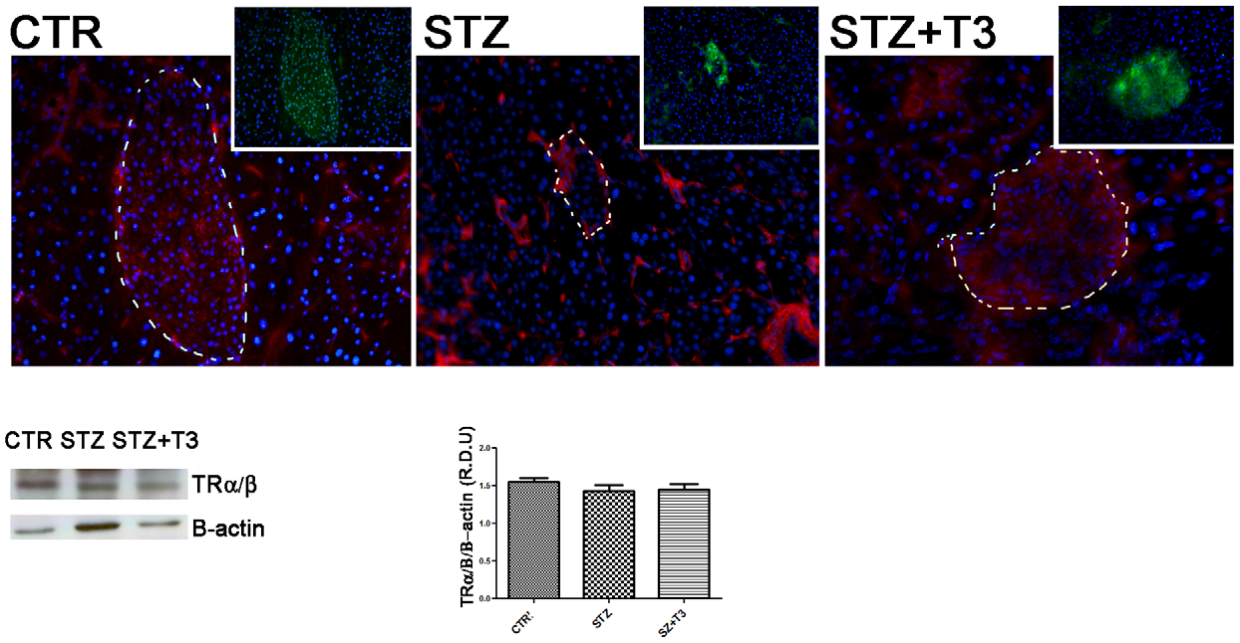
Thyroid hormone receptors expression. Tissue sections from the different experimental groups of animals have been obtained as described in the Materials and Methods section. Indirect immunofluorescence for Thyroid Receptor (Red) and Insulin (Greeen) revealed the presence of TR α/β within the islets and the pancreatic tissue surrounding. Nuclei were counterstained with Hoechst (blue). Data are from 1 or 2 experiments with similar results (n = 5 animals/group). At least ten fields *per* chamber and three independent cultures were examined. Space bar: 100 µm. Western Blot analyses were performed as described in Materials and Methods and a specific band corresponding to the Thyroid Receptor α/β was detected. The expression of B-actin was analyzed as a control for gel loading. At least three different experiments were performed, and a representative one is shown here. Densitometric absorbance values from three separate experiments were averaged (± SD), after they had been normalized to B-actin for equal loading. Data are presented on the right of the Western Blot panel in the histogram as Relative Densitometric Units (y axis). The different experimental groups are indicated on the x axis. A comparison of the individual treatment was conducted by using Student's *t* test. p = 0.003.

**Figure 2:**
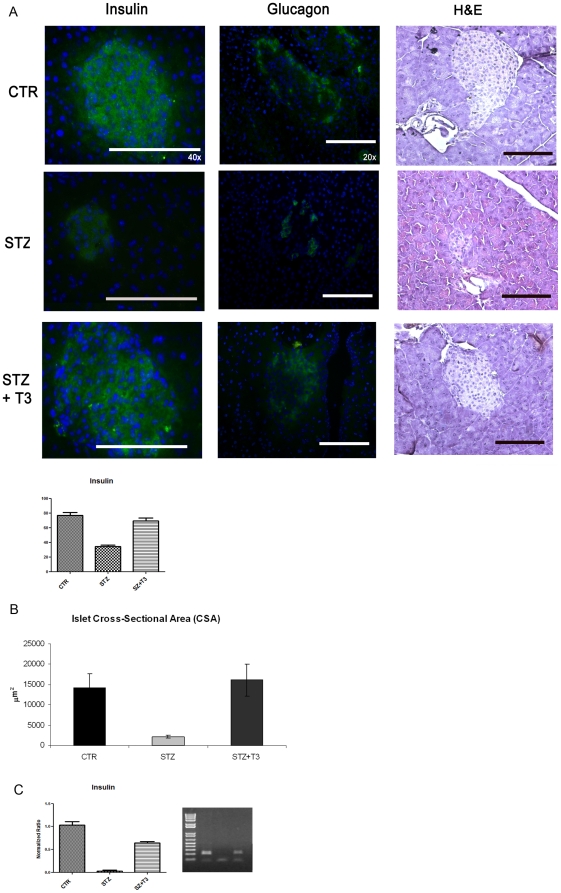
Islets analyses. **A** Histopathology: Tissue section from the different experimental groups of animals have been obtained as described in the Materials and Methods section. Immunofluorescence for Insulin (green, left panels), and Glucagon (green, middle panel), and Hematoxilin and Eosin staining (right panels) were performed to analyze histopathological changes in pancreatic islets compared to control mice (CTR). Nuclei were counterstained with Hoechst (blue) in the IF experiments. Data are from 1 or 2 experiments with similar results (n = 5 animals/group). At least ten fields *per* chamber and three independent cultures were examined Space bar: 100 µm. Histogram: The percentage of Insulin positive cells was calculated by counting up to a minimum of 200 cells for ten optical fields (200×) for each sample, randomly taken from two different experiments. The effect of treatment with T3 was statistically significant versus STZ. Student's *t* test: p<0.05 vs STZ+T3. **B** Islet crossection area: Crossection area was calculated as described in the Materials and Methods and the results were averaged and represented on the histogram. The presence of T3 significantly counteracts the reduction of islet area and deterioration. At least 10 different islets per sample were analyzed for each experiment. Data are from 1 or 2 experiments with similar results (n = 5 animals/group). The effect of treatment with T3 was statistically significant versus STZ+T3. Student's *t* test: p<0.01 vs STZ+T3. **C** Real Time PCR: Total RNA was obtained from pancreata from animals of the various experimental groups and RT-qPCR was performed as described in the Materials and Methods section. Melting point analysis of PCR products for both genes demonstrated single product formation, as confirmed by gel electrophoresis (on the right). All PCR products were of the expected size and sequence. Normalized ratios are shown in the histogram; the presence of T3 was able to overcome the STZ inhibition of Insulin gene expression.

To study the *in vivo* effects of the thyroid hormone T3, we used Balb/c mice. Diabetes has been induced with a “multiple low dose Streptozotocin injection protocol”, as described in the Materials and Methods section. The thyroid hormone action is mainly mediated through two thyroid receptor isoforms, namely TRα and TRβ. Our previous data evidenced that T3 action on pancreatic β cells and islets is predominantly mediated through the thyroid receptor β1 [19]. Hence even if the expression of the thyroid hormone receptors α and β in murine pancreatic islets has already been described [21], we confirmed this in our model by immunofluorescence and western blot analyses (Fig. 1). Mice were divided into three separate groups of study, namely the control one (CTR), which received vehicles alone; the Streptozotocin one (STZ), which received 2 intraperitoneal injections of STZ every 24 hours for 2 days; and the Streptozotocin+T3 one (STZ+T3), which received both T3 and STZ, ip every 24 hours, for 2 days. At the end of treatments, mice were sacrificed, and pancreata were excised. Size and shape of the islets were evaluated by hematoxylin/eosin staining, while the expression of insulin as well as of glucagon by immunofluorescence analysis (Fig. 2A). At the morphological level, as expected, islets from STZ-treated mice showed disarray of cellular architecture, irregular boundaries, reduced area and diminished β cell mass, when compared to control mice (Fig. 2A).

By contrast, T3 administration significantly prevented the decrease in β cell mass and islets area, counteracting the morphological changes induced by STZ. The β cell mass was directly analyzed by morphometry and the crossection area of the islets was measured as described in the Materials and Methods section. In fact, as shown in Figure 2B while the area of the Streptozotocin islets was significantly reduced, the decrease was completely overcome by the T3 treatment. Moreover, the T3-induced preservation of islets shape and size was not due to growth of undifferentiated mass, since no increase in the number of Ki67+ cells was observed (data not shown), excluding an eventual T3 caused indifferentiated mass growth. To better highlight the beneficial thyroid hormone effects on β cell mass and function, Real Time RT-PCR for Insulin has been performed. Typical standard curves plotted by the Lightcycler were obtained for Ins and rRNA 18s (r = −1). Melting point analysis of PCR products for both genes demonstrated single product formation, as confirmed by gel electrophoresis. As shown in the histogram, (Fig. 2C) whether the presence of Insulin mRNA was barely detectable in the STZ treated pancreata, the thyroid hormone treatment was sufficient to maintain the Insulin gene levels comparable to control (65%), strongly overcoming the STZ inhibition.

### T3 counteracts STZ induced islets apoptosis

**Figure 3:**
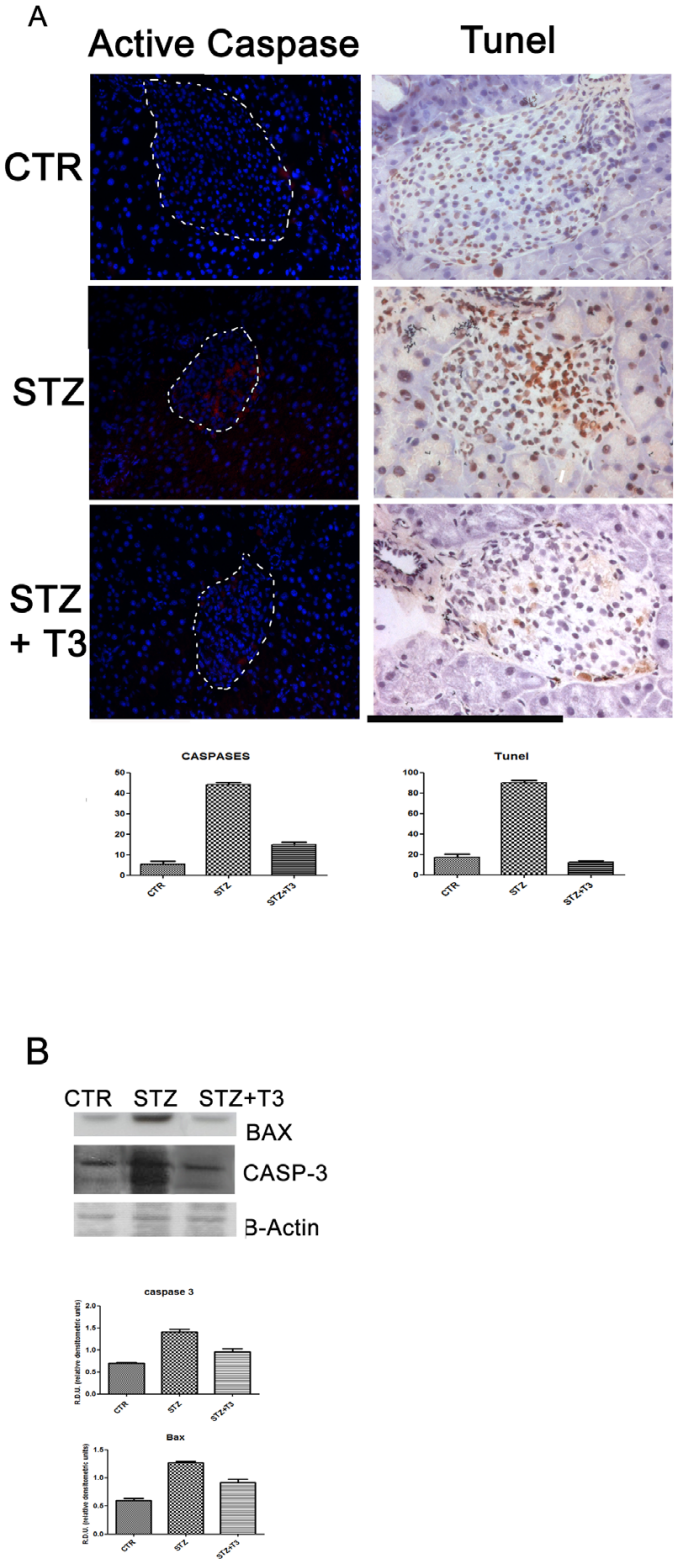
Apoptosis and survival. **A** Apoptosis measurement Tissue sections from the different experimental groups of animals have been obtained as described in the Materials and Methods section. Caspases activity (left panel, red) was detected by CaspGLOW and Tunel assay (right panel) was visualized by Immunohistochemistry. At 48 h of STZ alone apoptotic nuclei were clearly detectable within the islets, while in the samples exposed contemporary to T3 apoptotic nuclei were hardly detectable within the islets. At least 10 different islets per sample were analyzed for each experiment. Data are from 1 or 2 experiments with similar results (n = 5 animals/group). Space bar: 100 µm. Histogram: The percentage of Tunel or Caspase positive cells was calculated by counting up to a minimum of 200 cells for ten optical fields (200×) for each sample, randomly taken from two different experiments. The effect of treatment with T3 was statistically significant versus STZ. Student's *t* test: p<0.01 vs STZ. **B** Western Blot: Western Blot analyses were performed as described in Materials and Methods on protein extracts from the various experimental groups and a specific band corresponding to Bax and Casapase3 was detected. As shown, while the presence of STZ clearly induced the expression of Bax and the activation of Casp 3, the presence of T3 was able to counteract STZ action, maintaining Bax and Casp 3 levels comparable to the CTR samples. The expression of B-actin was analyzed as a control for gel loading. At least three different experiments were performed, and a representative one is shown here. Densitometric absorbance values from three separate experiments were averaged (± SD), after they had been normalized to B-actin for equal loading. Data relative to each protein are presented on the right of the Western Blot panel in the histogram as Relative Densitometric Units (y axis). The different experimental groups are indicated on the x axis. A comparison of the individual treatment was conducted by using Student's *t* test. p = 0.003.

Based on our previous observations in β cell lines and in *ex vivo* cultured islets, where the presence of T3 was sufficient to completely overcome the ongoing apoptotic process, and considering the evident preservation of the β cell mass in T3 treated mice, we wanted to verify whether T3 treatment could protect the animals from STZ-induced effects by preventing apoptotic cell death. To this aim, Tunel analyses and active caspases staining were assessed in pancreata from the different groups of mice. As shown in Figure 2, STZ treatment results in a much higher number of Tunel+ and active caspases+cells within the islets, when compared to control. These results demonstrate that the acute oxidative injury caused by STZ treatment induces a high level of cell death. By contrast, T3 co-treatment prevents STZ-induced cell death, lowering the number of Tunel+ and active caspases+cells to levels comparable to control samples, as shown in the histogram of Fig. 3A.

To deepen into the molecular changes induced by T3 in the apoptotic cascade, two major proapoptotic molecules have been analyzed by Western Blot, which are moreover both targeted by the Akt action. As shown in the Figure 3B, while STZ clearly induced BAX expression, the T3 can maintain its levels comparable to the control ones. In agreement, even the activation of the caspase 3, a Bax downstream, which is clearly evident and strong in the STZ samples, shows basal levels in both the control and the STZ+T3 ones, indicating that T3 can contrast its activation by STZ.

### T3 does not influence Glut-2 expression or localization

**Figure 4:**
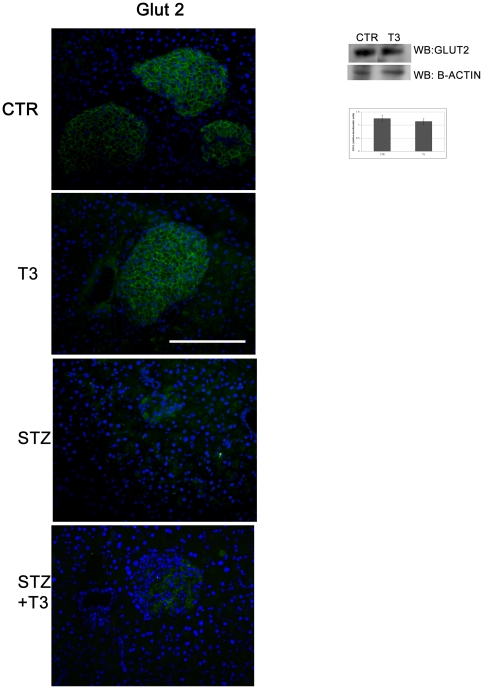
Glut-2 status. Immunofluorescence: Tissue sections from the different experimental groups of animals have been obtained as described in the Materials and Methods section. Indirect Immunofluorescence for Glut-2 (green) revealed the presence of the Glucose transporter within the islets, clearly detectable. Nuclei were counterstained with Hoechst (blue). As shown with T3 treatment, no differences either in the expression levels or in the localization of the Glucose transporter Glut-2 was observed. On the other hand, the presence of the STZ was sufficient to cause a strong disarray in both expression and localization of Glut-2 in the islets. Data are from 1 or 2 experiments with similar results (n = 5 animals/group). At least ten fields *per* chamber and three independent cultures were examined. Space bar: 100 µm. Western Blot: Western Blot analyses were performed as described in Materials and Methods on protein extracts from the CTR and the T3 treated animals and a specific band corresponding to Glut-2 was detected. The presence of T3 did not provoke any change in the Glut-2 expression, as compared to B-actin expression. The expression of B-actin was analyzed as a control for gel loading. At least three different experiments were performed, and a representative one is shown here. Densitometric absorbance values from three separate experiments were averaged (± SD), after they had been normalized to B-actin for equal loading. Data are presented on the right of the Western Blot panel in the histogram as Relative Densitometric Units (y axis). The different experimental groups are indicated on the x axis. A comparison of the individual treatment was conducted by using Student's *t* test. P<0.003.

Since it is known that the glucose-analogue STZ requires the β cell glucose transporter Glut-2 to enter the cell and to exert its apoptotic effect [22], we investigated Glut-2 expression and localization in the islets by immunofluorescence analysis. As shown in Figure 4, the STZ could completely disarray the Glut-2 expression and localization, consistently with its negative effect on the whole islet structure; on the other hand both control and STZ+T3 islets showed good localization and levels of the Glucose transporter. Interestingly, T3 treatment did not alter either expression or localization of Glut-2, ruling out the possibility that T3 could counteract apoptosis in β cells by preventing STZ entry via Glut-2. In addition, since Glut-2 is the key responsible in the glucose uptake by the β cell, we can hypothesize that T3 effects are not due to any alteration in the glucose entry in the cells.

### T3 preserves ultrastructure of β cells against STZ

**Figure 5:**
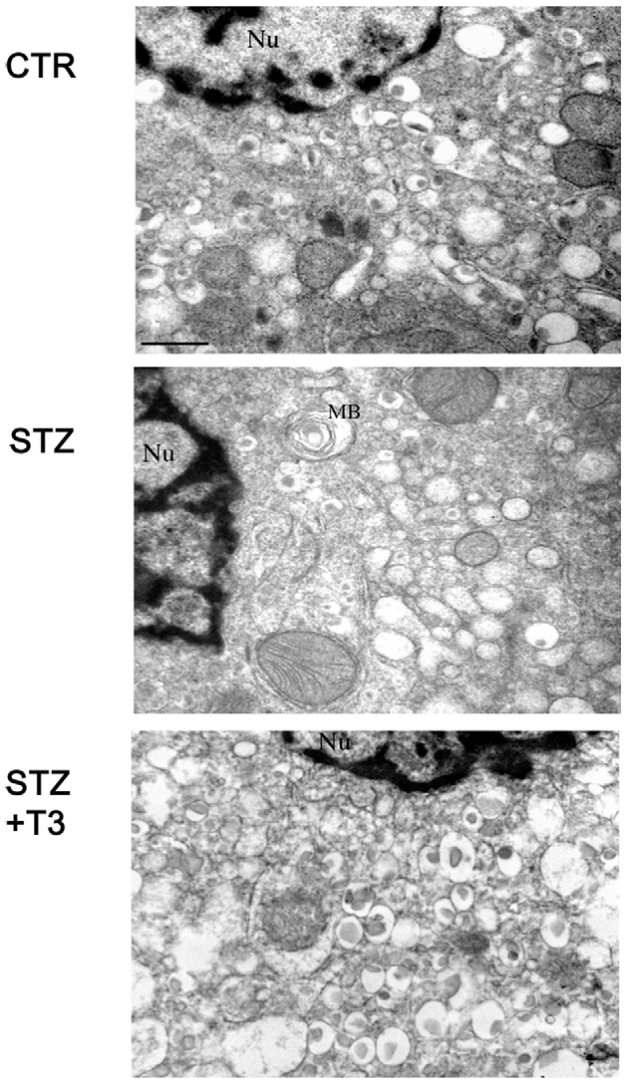
Ultrastructure of β cells. Trasmission electron micrographs of STZ and STZ+T3 treated pancreatic islets compared to control (Uranyl acetate/lead citrate; space bar 1 µm). Nu, nucleus; MB, myelinic bodies. The ultrastructure of the β cells was affected by STZ and maintained unaltered by the addition of T3. Data are from 1 or 2 experiments with similar results (n = 5 animals/group).

To further evaluate the morphological recovery induced by T3 treatment in STZ-treated mouse islets, TEM analysis was performed. Figure 5 contrasts the difference in ultrastructural appearance of well-granulated β cells in an islet of STZ+T3 mouse, as well as a CTR mouse, versus the extensively degranulated appearance of an islet from a STZ mouse. The marked reduction in insulin storage granules was generally associated with a dilatation of the rough endoplasmic reticulum. β cells containing mitochondria with a less dense matrix and partly damaged cristae were also observed, while large vacuoles, swollen cisternae of endoplasmic reticulum and myelinic bodies were also evident. These ultrastructural features are consistent with extreme secretory stress placed on residual β cells. In contrast, the β-cells of STZ+T3 treated islets were characterized by an overall unaltered ultrastructural morphology, which was similar to control islets. The cytoplasm contained numerous granules of the round medium-dense core type or with angular or round crystalline material surrounded by a large clear halo.

### T3 stimulates islets Akt activation

**Figure 6:**
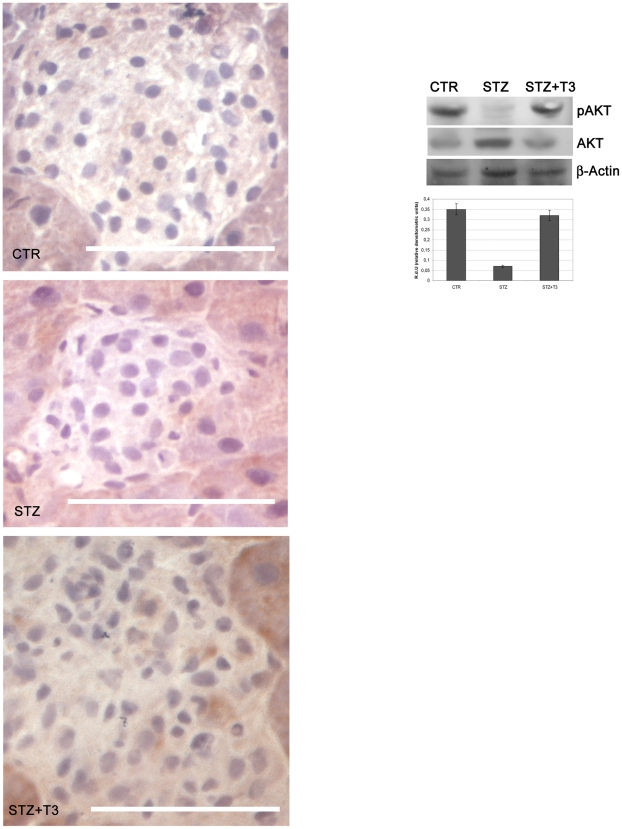
Akt activation. Immunohistochemistry: Tissue section from the different experimental groups of animals have been obtained as described in the Materials and Methods section. Immunohistochemistry for pAKT (Ser 473) was performed as described in the Materials and Methods. The presence of T3 clearly provoked an increment in Akt activation, as compared to total Akt expression (data not shown) Data are from 1 or 2 experiments with similar results (n = 5 animals/group). At least ten fields *per* chamber and three independent cultures were examined Space bar: 100 µm. Western Blot: Western Blot analyses were performed as described in Materials and Methods and a specific band corresponding to the phosphorylated Akt (Ser 473) was detected. The expression of total Akt was analyzed as a control for gel loading. The presence of T3 clearly provoked an increment in Akt activation (Ser 473), as compared to total Akt expression. At least three different experiments were performed, and a representative one is shown here. Densitometric absorbance values from three separate experiments were averaged (± SD), after they had been normalized to Akt for equal loading. Data relative to each protein are presented on the right of the Western Blot panel in the histogram as Relative Densitometric Units (y axis). The different experimental groups are indicated on the x axis. A comparison of the individual treatment was conducted by using Student's *t* test. P<0.001.

Previously, we demonstrated that T3 is able to activate the Akt signalling in pancreatic β cells and, most importantly, that this activation is the key event in the T3 action on pancreatic β cell function and survival; hence, we sought to verify whether the observed T3-induced anti-apoptotic action may depend on Akt activation/phosphorylation. As shown in Figure 6, immunostaining for pAkt (Ser473) demonstrated that, while in STZ-treated islets Akt activation was significantly inhibited, when T3 was additionally administered, the levels of Akt phosphorylation were actually maintained comparable to control, untreated islets. This observation was further confirmed by Western blot analysis (Fig. 6, right panel). Furthermore considering that Akt survival action includes the regulation of some proapoptotic factors, as Bax and caspase 3, the evidence for Akt activation can easily be related to the shown Bax and Casp 3 inhibition by T3.

These results, together with our previous evidences in β cell lines and in islets, strongly suggest that T3 exerts its β cell protective effect at least in part through the Akt signalling.

### T3 preserves glucose responsiveness in STZ treated mice

Metabolic parameters were assessed in mice. After STZ injection mice were diabetic with significant fasting hyperglycemia, as described below, glycosuria and hypoinsulinemia when compared with age-matched control mice and STZ+T3 mice. At the time of sacrifice body weight was still similar between mice injected with either STZ or STZ+T3, showing a little decrease only in few case of STZ injected animals. This was presumably due to dehydration and protein wasting associated with diabetes. Thus, the STZ injection protocol we used generated an experimental model of type 1 diabetes, as expected.

**Figure 7:**
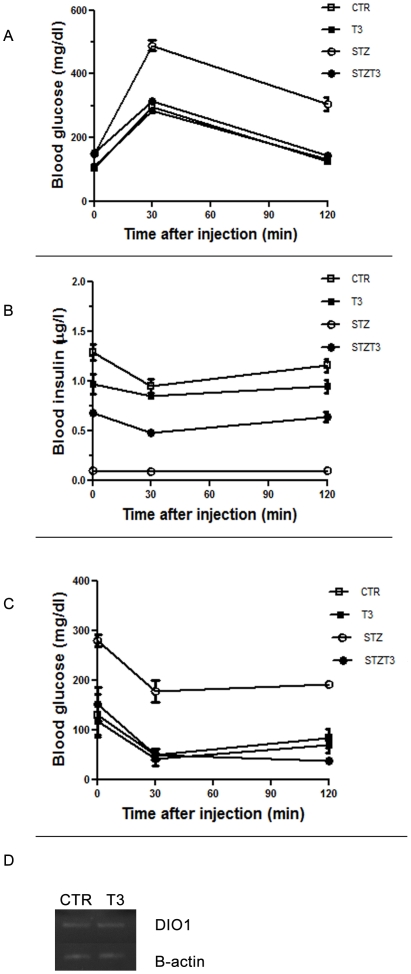
Phisiological parameters. **A, B**: Analysis of blood glucose and Insulin levels after intra-peritoneal glucose tolerance test (upper panels). Glycemia was measured by glucometer, while Insulin concentration was assessed by ELISA assay, as described in the Materials and Methods section. Oral administration of T3 significantly reduces severity and progression of STZ-induced diabetes in Balb/c mice and assured normal Insulin responsiveness. **C**: Insulin tolerance was performed (lower panel) after intra peritoneal glucose injection. Insulin was injected intraperitoneally after glucose to the different experimental groups of animals. Glycemia was measured by glucometer. Results represent the mean ± SE of three separate experiments. Grey: control black:STZ white:STZ+T3. **D** RT-PCR: Total RNA was extracted from liver from mice of the different experimental groups and RTPCR was performed as described in the Materials and Methods section. A single product was obtained for each gene, as showed by agarose electrophoresis. All PCR products were of the expected size and sequence. The presence of T3 did not induce any change in the DIO1 expression, as normalized to 18s.

Considering that T3 increases β cell function and survival, as we previously demonstrated, and that it maintains β cell mass in STZ-treated mice, it is conceivable that T3 may act as an anti-diabetic factor, ensuring euglycemic status by preserving β cell mass. We thus analyzed the ability of the different groups of mice to respond to an ip glucose tolerance test. Among the 25 mice receiving STZ, 21 became overlay diabetic (blood glucose >250 mg/dl); while 4 showed borderline elevated blood glucose (300>200 mg/dl). In contrast, among the 25 mice treated with T3 together with STZ, only 4 became diabetic; the remaining 18 maintained normal blood glucose levels (<150 mg/dl), while 3 died. In addition, glucose tolerance test evidenced that while the Streptozotocin treated mice completely lost their ability to normally respond to glucose loading, the presence of T3 preserves the ability of mice to restore their normal glycemia 120 min after glucose loading and maintains the serum glucose levels in the euglycemic range (Fig. 7).

### T3 preserves islets function in STZ treated mice

Finally, we assayed serum insulin levels to analyze the effect of T3 treatment on islets function. As shown in Figure 7 (A), STZ treatment induced a significant decrease in the insulin response, as showed by the lower levels of serum insulin at the different time points, according to the affected ability of control glucose blood levels; on the other hand when T3 was administered at the same time of STZ, serum insulin levels were comparable to the control (Fig. 7B), suggesting that T3 treatment preserves insulin production, preventing STZ effects. These final observations supported the hypothesis on that T3 acts as an antidiabetic *in vivo*, preserving β cell mass, counteracting β cell apoptosis and regulating the insulin response, via the Akt signalling.

To better characterize the physiology of our mice, we decided to exclude the occurence of Insulin intolerance by an Insulin Tolerance test. As shown in the histogram in Figure 7C, all animals showed an adequate Insulin responsive, although, as expected, glucose blood levels were higher in the animals treated with STZ.

In all the experiments described above, the serum levels of FT4 and FT3 were evaluated by chemiluminescence to exclude the presence of hyperthyroidism in the animals; moreover the expression of the deiodinase 1 in liver was not altered by the treatment, as shown in Figure 7D, indicating a condition of euthyroidism in the T3 treated mice.

## Discussion

In this paper we describe a novel protective action of thyroid hormone T3 from STZ-induced diabetes *in vivo*. Although recent clinical evidences indicate that thyroid hormone treatment can ameliorate diabetic condition [26], [27], our study is the first one to focus on β cell function in a diabetic animal model (STZ-treated mice) in the presence of T3 administration.

At first, T3 treatment prevented STZ-induced islets deterioration, as shown by the maintenance of the islet structure, size and consistency. Indeed, while STZ treatment induced reduction in islets size and cell number, the morphology of the islets, the abundance and distribution of insulin-, as well as glucagon-, expressing cells in the animals treated with T3 and STZ, remained comparable to islets derived from control mice. Preservation of islets morphology was also confirmed at the ultrastructural level, where the presence of T3 prevented the induction of STZ-induced features of cell damage (clumped chromatin, disorganized insulin-containing granules, altered mitochondria, endoplasmic reticulum and vacuoles morphology). The observed ability to preserve the islets appearance was associated with a protective role of T3 on STZ-induced β cell death, as shown by both Tunel analysis and caspases activation. The STZ-induced cell death observed within the islet was almost completely prevented by T3. It is known that Streptozotocin enters β cells via the glucose transporter Glut-2 and induces islets deterioration by inducing β cells apoptosis [22]. We sought that neither expression or localization of Glut-2 was altered by T3 treatment, ruling out that the observed effects may be dependent on impairment in Streptozotocin internalization. These results are consistent with our previous observations where we showed that T3 may be considered a mitogenic and survival factor for pancreatic β cells *in vitro* : it preserved, indeed survival and function in freshly isolated islets in culture and protected cultured β cells from pharmacological induced apoptosis [17], [19]. Taken together our results show that the main mechanism leading to the increase in β cell mass, survival and function, when T3 is administered together with STZ, is based on the prevention of STZ-induced β cell apoptosis.

Increasing evidences indicate that the decrease of the functional β-cell mass is the hallmark of both Type 1 and Type 2 diabetes, resulting in the absolute or relative insulin insufficiency in both conditions. In this context, β-cell apoptosis and impaired proliferation, consequences of hyperglycemia, are features that may be present in all forms of diabetes, suggesting that the classification of diabetes should be revalued [23], [24], [25]. β cell death can thus be considered as the key event of such diseases, highlighting the urgence to identify factors able to specifically target the β cell mass, avoiding any β cell toxic side effects.

The PI3K/Akt pathway is a common pathway activated by a variety of growth factors to stimulate cell growth and survival. In particular, Akt activation has been shown to induce β cell proliferation, survival, mass and function [5], [28]. Thus, factors able to stimulate Akt have gained relevance in the search of new anti-diabetic strategy. We showed in this paper that the observed T3-induced anti-apoptotic effects are associated with activation of the Akt signalling pathway in the islets. This is consistent with recent data showing the ability of the thyroid hormone to stimulate Akt in neurons [29], in vascular myocytes [30] and in other cell types [31]. In addition, we previously demonstrated that T3 stimulates Akt in pancreatic β cells *in vitro* [18], leading to the activation of mTOR, GSK3B, B-catenin and others [19]; in the present study we did not deepen into the Akt pathway, but considering our *in vitro* data, it is conceivable that the survival action of T3 in mice might involve mainly the same mechanisms. The important outcome of the observed T3 protective effects in β cell survival and function is the preservation of pancreatic metabolic activity. Indeed, we showed that T3 administration actually preserves an intact response to glucose, and keeps plasma insulin levels in STZ-treated mice comparable to those in control mice; moreover, we showed that both STZ and STZ+T3 treated mice do not develop insulin resistance.

While β cell loss by apoptosis is a recognized feature of both type 1 and type 2 diabetes, approaches to block this process are limited, so far. Currently, the main goal for diabetes treatment is the maintenance of glucose homeostasis as close to normal as possible in order to avoid the devastating complications of this disease. These treatments include oral hypoglycemics and insulin sensitizers, different insulin preparations administered daily by multiple injections, continuous insulin pumps and, in some T1D patients, transplantation of the whole pancreas or islets. None of these approaches is focused on the maintenance of endogenous β cell mass, though it has been shown that even a small amount of preserved endogenous insulin secretion has great benefits in terms of clinical outcome [32]. Therefore, finding a molecule that could be useful to block β cell apoptosis and thereby preserve and enhance endogenous β cell mass would represent a major breakthrough. The results presented in this study suggest that T3 may actually be a good candidate.

To this aim, however, therapeutic protocol should be accurately designed, in terms of both doses and time intervals, to avoid side effects. It is known indeed that excess of thyroid hormones production by the thyroid gland or by exogenous thyroid hormones administration, results in hyperthyroidism or thyrotoxicosis, characterized by tachycardia, with possible atrial arrythmias and heart failure, muscle wasting, osteoporosis in post-menopausal women, and other symptoms [33]. However, thyroid hormone excess also results in beneficial effects, including the metabolic ones. Given the widespread effects of thyroid hormones on the physiology of multiple organs, the chance to use them in a therapeutical fashion remains attractive. In this context, it has been recognized that the induction of a subclinical hyperthyroidism, especially if temporary induced, might be well tolerated by patients and could be accepted in some clinical cases. As reported by Kaptein et al [34], thyroid hormone treatment in obese patients with nonthyroidal illnesses provoked only a subclinical risk and no significant side effects concerning either weight loss or heart rate; neither mortality was worsened by THs treatment. In the present study, animals, which received thyroid hormone T3 for 48 h, did not show any significant risky alterations in the thyroid status and did not develop any hyperthyroidism.

Next to the possibility to use T3 (or analogues) *in vivo* to counteract diabetes, given the pro-survival and antiapoptotic activity on β cells described, T3 administration may also be considered to improve setting for islets transplantation. A major impediment indeed to islet transplantation is the large number of islets required in order to confer insulin independence, resulting in the need of several organ donors [35]. This fact is in sharp contrast to the known small amount of β cell mass necessary for the mainteinance of glucose homeostasis *in vivo*. It is therefore assumed that a large fraction of transplanted islets undergoes apoptosis and is lost. We recently demonstrated that the administration of T3 to the islets in culture preserves their vitality against both physiological and pharmacological cell death. T3 treatment makes islets less susceptible to stress during the transplantation, preventing β cell loss, reducing the number of the required islets and thereby improving the outcome of islets transplantation.

In conclusion, our findings demonstrate for the first time that T3 administration counteracts STZ-induced diabetes, as being a pro-survival, anti-apoptotic factor for β cells, and thus preserves glucose sensing machinery. Altogether these results suggest that T3 can be considered or added to diabetes therapy.

As both type 1 and type 2 diabetes are diseases where deficiency in β cell mass and function is pathogenic, the ability of thyroid hormone to preserve islet mass without loss of β cell differentiated function makes T3 an attractive factor for future therapies for diabetes.
